# Acute and Chronic Effects of N-acetylcysteine on Pentylenetetrazole-induced Seizure and Neuromuscular Coordination in Mice

**Published:** 2015-03

**Authors:** Sasan Zaeri, Masoumeh Emamghoreishi

**Affiliations:** 1 Department of Pharmacology, School of Medicine, Shiraz University of Medical Sciences, Shiraz, Iran;; 2 Department of Neuroscience, School of Advanced Medical Sciences and Technologies, Shiraz University of Medical Sciences, Shiraz, Iran

**Keywords:** N-acetylcysteine, Pentylenetetrazole, Anticonvulsant, Seizure

## Abstract

**Background:**

N-acetylcysteine (NAC) has been indicated against experimental seizures, but with relatively inconclusive results. This study was undertaken to evaluate whether NAC exerts a dose-dependent anticonvulsant effect and to determine NAC safe therapeutic dose range and its muscle-relaxant activity in both acute and chronic uses.

**Methods:**

Following intraperitoneal (i.p.) administration of N-acetylcysteine acutely (50-300 mg/kg) or chronically for 8 days (25-300 mg/kg), mice were injected with PTZ (90 mg/kg, i.p.) and latency times to the onset of myoclonic and clonic seizures and protection against death were recorded. Changes in body weight and mortality rate were considered as parameters for drug safety. The muscle-relaxant activity of NAC was assessed by rotarod test.

**Results:**

Acute and chronic treatment with NAC delayed latency times to myoclonic and clonic seizures in a dose-dependent manner, but with no significant prevention against PTZ-induced death. Chronic administration of 300 mg/kg NAC was fully lethal while lower doses (100 and 150 mg/kg) resulted in a significant weight loss and decreased stay time on rotarod. Acute treatment with NAC had no significant effect on stay time on rotarod at all studied doses.

**Conclusion:**

NAC exerts a dose-dependent anticonvulsant effect in acute and chronic uses, with no muscle relaxant activity. NAC has higher efficacy in preventing seizure in chronic than acute treatment, but its chronic use at higher doses of 75 mg/kg may be associated with side effects and/or toxicity. These findings suggest that low doses of NAC may have a potential use as a prophylactic treatment for absence seizure in human.

## Introduction

The exact pathophysiological basis of epilepsy is currently unknown. However, recent evidence implicated an important role for oxidative stress in the pathophysiology of neurological disorders, including epilepsy.^1^^-^^3^ Thus, a number of insults that make brain prone to seizure, such as hypoxia, trauma, and aging^4^^-^^7^ can produce oxidative stress and mitochondrial dysfunction. Furthermore, the process of oxidative stress has been implicated in several rodent models of seizures including pentylenetetrazole (PTZ)-induced seizure.^8^^-^^10^ Significant decreases in thiols concomitant with significant increases in thiol disulfides, lipid peroxidation and protein oxidation have been reported in hippocampus of PTZ-treated mice.^10^ Taken together, the free radical production and stress oxidative processes have been indicated in the development of seizures.

Current anticonvulsant drugs are largely aimed at decreasing neuronal excitability to prevent or control the occurrence of epileptic seizures. However, the role of oxidative stress processes in seizures led to the proposition that antioxidant compounds may be considered as promising candidates for preventing the development of epilepsy.^11^ Accordingly, several antioxidant compounds have been examined against experimental seizures.^12^^-^^14^

N-acetylcysteine (NAC), a well-known antioxidant agent with both peripheral and central antioxidant activity,^10^ may be a candidate with possible anti-seizure activity. However, experimental findings on anti-seizure effect of NAC has been confined to a few studies which have mainly used NAC to elucidate the role of oxidative stress and free radical generation in induction of seizure, or to evaluate its possible effect on decreasing toxicity or facilitating anti-seizure effects of current anticonvulsants as adjuvant therapy.^8^^,^^15^^-^^18^ These studies were not designed to determine the anticonvulsant activity of NAC per se and thus used only one or two doses of NAC either acutely or chronically. Therefore, the aims of this study were (i) to investigate whether NAC has dose-dependent anticonvulsant effect; (ii) to determine whether the anticonvulsant activity of NAC sustains after long-term usage without toxicity by evaluating the acute and chronic effects of NAC on PTZ-induced seizure and on weight and mortality of animals; and (iii) to determine whether possible muscle relaxant activity of NAC may contribute to its proposed anticonvulsant property by evaluating the effect of NAC on neuromuscular coordination. The findings of the present study broaden our understanding of the anti-seizure effect of NAC and offer its future potential use in absence seizure in human.

## Materials and Methods

*Animals*

This study was approved by the Ethics Committee of Shiraz University of Medical Sciences for animal care. 

Male albino mice (25-35 g) (N=105) were housed in polypropylene cages with free access to water and food and under controlled light/dark cycle (12/12 h) and temperature (22±2°C). All experiments were performed between 8.00 am to 1.00 pm.

*Chemicals*

N-acetylcysteine, (Exir®, Iran) was dissolved in 0.05% EDTA in distilled water and kept in the dark. Pentylenetetrazole (K&K, USA), diazepam (10 mg/ml) and Ethosuximide (ChemiDaru®, Iran) were dissolved in normal saline. All solutions were freshly prepared and were intraperitoneally (i.p.) injected in a volume of 0.1 ml per 10 g animal weight.

*Experimental Procedures*

*Evaluation of Acute Effect of NAC on PTZ-induced Seizure*

Forty-nine mice were randomly distributed in seven groups (7 per group). The groups received a single dose of 50, 75, 150 or 300 mg/kg of NAC, diazepam (1 mg/kg) as a reference drug, EDTA 0.05% in distilled water (vehicle for NAC) or normal saline (vehicle for diazepam). The doses of NAC were chosen based on a pilot study at our laboratory and a previous study.^19^

*Evaluation of Chronic Effect of NAC on PTZ-induced Seizure*

Fifty-six mice were randomly allocated to eight groups (7 per group). Mice were administered NAC (25, 50, 75, 100 or 150 mg/kg; i.p.), ethosuximide (150 mg/kg; i.p.) as a reference drug, EDTA 0.05% in distilled water (control for NAC), or normal saline (control for ethosuximide), once daily for 8 days. In a pilot study, chronic administration of 300 mg/kg NAC resulted in weight loss and consequent death, therefore, this dose was excluded from the chronic study. 

*PTZ-induced Seizure*

Mice were administered PTZ (90 mg/kg, i.p.), one hour after receiving either a single dose (acute treatment) or 8-day multiple doses (chronic treatment) of drug or vehicle. This dose of PTZ produced myoclonic and clonic seizures followed by death in 100% of animals in a control group. Latency times to the onset of myoclonic and clonic convulsions were recorded during 30 minutes after the injection of PTZ. The percentages of mortality were also recorded up to 24 hours after PTZ injection. The latency time was reported as 1800 seconds when a mouse did not show any kind of seizure within the cut-off time. 

*Evaluation of Acute and Chronic Effects of NAC on Neuromuscular Coordination*

The day before the experiment, mice were trained to stay on a rotating rod (3 cm diameter, 20 rpm) for at least 120 seconds.^20^ In acute study, mice were tested on rotarod (UGO Basil, Italy) before and 50 minutes after the administration of NAC or vehicle. In chronic study, mice were tested before the first injection of the drug or vehicle on the first day and 50 minutes after the last injection on the 8^th^ day of treatment. Latency time to fall from the rotating rod was recorded for a maximum of 300 seconds.

*Statistical Analysis*

The data were analyzed by SPSS^®^ (version 16.0) and presented as mean±SEM. Parametric tests were used for statistical analyses as Kolmogorov-Smirnov showed normal distribution of the data. Latency times were analyzed by one-way ANOVA followed by Tukey’s test. Univariate analysis of variance was performed for the rotarod data with baseline values as covariate. Independent *t *test was used to analyze mean differences between diazepam or ethosuximide and their respective controls. The percentages of mortality were analyzed by Pearson Chi square test. Linear regression was used to assess the dose dependency of NAC effects. A value of P<0.05 was considered significant.

## Results

*Acute Effect of NAC on PTZ-induced Seizure*

**Figure 1:**
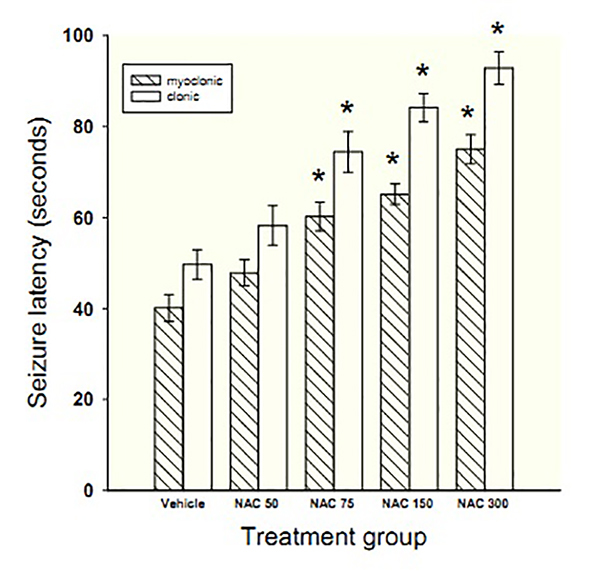
Bars represent mean±SEM of latency times to the onset of myoclonic and clonic seizures for 7 mice/group following acute treatment with different doses of NAC (50-300 mg/kg) or vehicle. *Indicates significance (P<0.05) in comparison with vehicle

NAC at doses of 75, 150 and 300 mg/kg significantly increased latency time to the onset of myoclonic seizure by 50, 62 and 87% (F=22.7, P<0.001) and clonic seizure by 49, 69 and 86% (F=22.07, P<0.001), respectively as compared with the vehicle group (figure 1). Diazepam protected all animals from myoclonic and clonic seizures within the 1800 seconds of observation (data not shown).

Linear regression demonstrated the dose-dependent effect of NAC on prolonging time to myoclonic (slope=0.11, P=0.002) and clonic (slope=0.14, P=0.002) convulsions. In addition, Tukey’s post hoc test showed significant differences in latencies to myoclonic and clonic seizures between adjacent doses of NAC (P<0.001). 

NAC at doses of 50, 75, and 150 mg/kg had no protective effect against PTZ-induced death, but protected 14% of the animals from death at 300 mg/kg. Diazepam protected all animals from PTZ-induced death.

*Chronic Effect of NAC on PTZ-induced Seizure*

**Figure 2:**
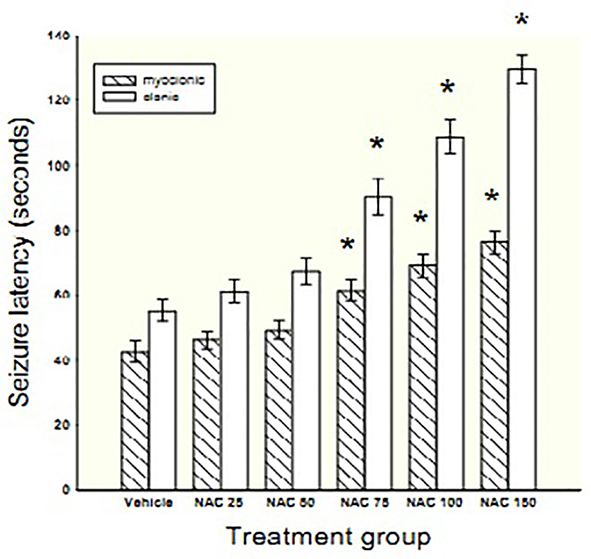
Bars represent mean±SEM of latency times to the onset of myoclonic and clonic seizures for 7 mice/group following chronic treatment with different doses of NAC (25-150 mg/kg) or vehicle. *Indicates significance (P<0.01) in comparison with vehicle

NAC at doses of 75, 100 or 150 mg/kg significantly prolonged the latency time to myoclonic seizure by 44, 62 and 79% (F=17.4, P<0.001) and clonic seizure by 64, 97 and 135% (F=41.75, P<0.001), respectively in comparison with vehicle (figure 2). Ethosuximide protected all animals from myoclonic and clonic seizures within the 1800 seconds of observation (data not shown).

Chronic NAC treatment showed a dose-dependent increase in latency time to myoclonic (slope=0.25, P<0.001) and clonic (slope=0.54, P<0.001) seizures. In addition, Tukey’s post hoc showed significant differences in latencies to myoclonic and clonic seizures between adjacent doses of NAC (P<0.001).

Chronic treatment with NAC at doses of 25 and 50 mg/kg had no protective effect against PTZ-induced death, but at doses of 75, 100 and 150 mg/kg protected 14, 14 and 33% of animals from death, respectively. Ethosuximide protected 100% of animals against PTZ-induced death.

Univariate analysis of variance with baseline weight as covariate followed by the Dunnett’s t-test revealed that animals receiving 100 and 150 mg/kg of NAC significantly lost weight in comparison to vehicle group (F=3.76, P*=*0.008).

*Acute and Chronic Effects of NAC on Neuromuscular Coordination*

Acute doses of NAC (50-300 mg/kg) had no significant effect on stay time on rotarod in comparison to vehicle (F=0.40, P=0.805) (table 1). Diazepam significantly decreased the stay time on the rotarod in comparison to control (P<0.001).

**Table 1:** The effects of acute and chronic treatment with different doses of NAC on neuromuscular coordination

**Treatment group**	**Dose (mg/kg)**	**Acute** **stay time on rotarod (s)**		**Chronic** **stay time on rotarod (s)**	
		**Baseline**	**Test**	**Baseline**	**Test**
Vehicle (EDTA)	-	250.6±21.9	259.4±18.1	244.6±20.1	255.4±16.6
NAC	25	-	-	230.1±26.5	229.5±23.1
	50	222.7±24.8	251.4±26.4	237.0±25.9	226.4±25.1
	75	244.7±24.4	254.4±26.1	231.6±25.2	180.5±20.9
	100	-	-	250.3±20.1	166.5±21.2*
	150	246.0±21.1	249.3±23.1	233.7±23.0	61.6±15.6*
	300	233.0±26.0	250.8±21.15	-	-
Diazepam	1	225.1±22.4	103.7±18.1*	-	-
Ethosuximide	150	-	-	244.3±23.1	236.7±20.2

Data were presented as mean±SEM of stay time on rotarod (n=7/group) before (baseline) and after (test) the injection of different doses of NAC, vehicle, diazepam or ethosuximide. *Indicates significance (P<0.01) in comparison with vehicle

There was a significant effect of chronic treatment on stay time on rotarod (F=14.02, P<0.001). NAC at doses of 100 and 150 mg/kg significantly reduced the time mice spent on the rotarod by 35 and 76%, respectively, as compared with vehicle (P=0.040 and P<0.001, respectively) (table 1). Doses of 25, 50 and 75 mg/kg NAC and ethosuximide had no significant effect on stay time on rotarod, as compared with control.

## Discussion

This study showed that NAC has a dose-dependent anticonvulsant effect, both in acute and chronic use, with no muscle relaxant activity. NAC had higher efficacy in preventing seizure in chronic than in acute treatment, but its chronic use at doses higher than 75 mg/kg was associated with weight loss and death in mice. 

Acute treatment with NAC showed dose-dependent comparable anticonvulsant effects against both clonic and myoclonic seizures in mice. No other studies have evaluated the acute effect of NAC against PTZ-induced seizure to be compared with our results. However, pretreatment with 200 mg/kg, but not 100 mg/kg, NAC prolonged latency time to aminophylline-induced seizure in mice.^16^ The current findings indicated that the anticonvulsant effect of NAC starts at a lower dose in animal model of PTZ- than aminophylline-induced seizure. This might be due to differences in the nature of seizure induction by PTZ and aminophylline. In another study,^18^acute treatment with fixed ratio combination doses of NAC and phenytoin or NAC and sodium valproate significantly controlled maximal electroshock (MES)-induced seizure in mice. However, they did not report the effect of NAC alone on MES-induced seizure in detail.

Our finding that chronic NAC has protective effect against PTZ-induced seizures is in agreement with a previous report by Devi et al.^8^ However, in contrast with our finding, they reported non-dose-dependent anticonvulsant effect of NAC. This discrepancy can be attributed to dissimilar experimental designs due to different objectives of the studies. Thus, in the present study, a single convulsive dose of PTZ (90 mg/kg) was used after 8 days of chronic treatment with 5 different doses of NAC, while in Devi et al. study a lower dose of PTZ (60 mg/kg) was used after chronic treatment with only two doses of NAC (50 and 100 mg/kg). The low dose of PTZ used in Devi et al. study was not probably enough to cause significant oxidative stress (as mentioned by the authors) to detect a dose-dependent anticonvulsant effect of NAC. In another study by the same group,^17^ neither 50 mg/kg nor 100 mg/kg of NAC showed anticonvulsive activity. Such controversy is probably due to the nature of the experimental seizure model used. In another study, chronic oral administration of NAC (100 mg/kg) controlled seizures induced by a subconvulsive dose of PTZ after a 5-week continuous exposure of animals to traumatic brain injury.^15^Though these results are in line with ours, they have not evaluated dose-dependency of NAC effects.

Acute and chronic administration of similar doses of NAC had alike effects against PTZ-induced myoclonic seizure. Instead, equivalent doses of NAC had more pronounced effects on clonic seizure in chronic than in acute treatment. This observation may be explained by the ability of NAC to take part in glutathione (GSH) synthesis. GSH has been reported to decrease following PTZ-induced seizures in experimental animals.^21^ NAC by providing cysteine can take part in replacing GSH in CNS. However, GSH synthesis requires enzymatic reactions with more than one enzyme involved,^22^ and enough time is likely needed for NAC to efficiently replenish GSH reservoirs in CNS. Therefore, NAC may increase GSH basal levels during chronic, but not acute, administration. This notion is supported by a report that NAC increased reservoir levels of GSH in the liver and kidneys during an 8-day treatment period in experimental animals.^8^However, this concept should be addressed in future studies measuring GSH levels in CNS following acute and chronic treatment with NAC.

The distinct effect of chronic NAC treatment on myoclonic and clonic seizures is difficult to explain. However, if oxidative stress has differential roles in the pathogenesis of myoclonic and clonic seizures, then NAC would be expected to exert different effectiveness in myoclonic and clonic seizures. Obviously, more studies are needed to clarify and confirm this likelihood.

The acute effect of NAC against PTZ-induced seizure may be described by its direct antioxidant activity. In fact, its free thiol group interacts with the electrophilic groups of ROS producing NAC disulfide as a major end-product.^23^ Moreover, NAC can increase the activity of catalase and SOD, important antioxidant enzymes, in aged rats.^24^ In addition, other antioxidant agents such as melatonin and vitamin C have also shown efficacy against experimental seizures.^12^^-^^14^ Abnormal neuronal discharge is the main characteristic of seizure. Several studies have suggested that ROS may increase cellular excitability and result in abnormal neuronal discharge by inhibiting Na^+^/K^+^-ATPase pumps.^25^ Accordingly, it seems that inhibition of free radicals by antioxidants, either directly or indirectly by increasing levels/activities of GSH, GSH peroxidase, SOD, or catalase could prevent abnormal neuronal discharge and in turn generation of seizure. However, the exact mechanism by which NAC acts against the process of oxidative stress to delay the generation of seizure should be elucidated in future studies.

Acute and chronic NAC treatments could not significantly protect animals from PTZ-induced death. This suggests that oxidative stress per se may not be the only factor involved in PTZ-induced death, but other parameters such as intensity and duration of seizure may play roles as well. These possibilities should be addressed in future studies.

Chronic high doses of NAC caused a significant reduction in stay time on rotarod. As NAC acutely had no muscle relaxant activity, this finding might be due to the muscle weakness caused by the weight loss observed after chronic NAC treatment. In support of this notion, there was a parallel correlation between weight loss and the stay time on rotarod in mice received NAC chronically (data not shown). This suggests that significant weight loss following chronic high doses of NAC likely disabled the animals to maintain on rotarod. No other studies has yet evaluated or reported weight loss following chronic treatment with different doses of NAC. The reason for such a weight loss is not clear. However, it may be related to NAC induced zinc-deficiency^26^^,^^27^ as anorexia has been reported to be the first symptom of zinc-deficiency in experimental animals.^28^

The present study showed that NAC by itself is effective against PTZ-induced seizure in mice in a dose-dependent manner. Compounds effective in PTZ model of seizure seem to be effective in petit mal seizure in human. Therefore, NAC may have a potential clinical use in petit mal seizure. This is supported by a report that NAC was found to improve clinical manifestations of a refractory type of myoclonic epilepsy.^29^ Chronic NAC showed anti-seizure effect in mice at a dose range of 75-150 mg/kg/daily which is equivalent to a daily regimen of 5.75-11.5 mg/kg in human (400-800 mg/day for a 70 kg individual ).^30^ This is within therapeutic doses of NAC as a mucolytic agent. Nevertheless, the optimum therapeutic doses of NAC as an anticonvulsant should be determined in future clinical trials.

This study was designed to achieve its objectives as mentioned above. However, the potential limitation of the study was that changes in oxidative stress parameters (GSH and antioxidant enzymes, e.g. SOD) were not measured in the brain of NAC-treated mice. This can be addressed in future studies to elucidate the mechanism by which NAC prevents seizure. In addition, as different experimental models of seizure represent different pathological processes and types of seizures, the use of other animal models such as MES is recommended in future studies. 

## Conclusion

NAC exerts a dose-dependent anticonvulsant effect, both in acute and chronic use. Chronic treatment with NAC has higher efficacy in preventing seizure than acute treatment, but its chronic use at higher doses of 75 mg/kg may be associated with side effects and/or toxicity. The results of this study propose that the anticonvulsant effect of NAC is not related to a muscle relaxant activity. The findings of this study suggest that low doses of NAC may have a potential use as a prophylactic treatment of absence seizure in human in the future. 
